# Chromosome-level genome assembly of Guide Black-Fur sheep (*Ovis aries*)

**DOI:** 10.1038/s41597-024-03564-x

**Published:** 2024-06-29

**Authors:** Zengkui Lu, Chao Yuan, Xuejiao An, Zhixiang Chen, Tingting Guo, Jianbin Liu

**Affiliations:** 1 Key Laboratory of Animal Genetics and Breeding on Tibetan Plateau, Ministry of Agriculture and Rural Affairs, Lanzhou Institute of Husbandry and Pharmaceutical Sciences, Chinese Academy of Agricultural Sciences, Lanzhou, 730050 China; 2 Sheep Breeding Engineering Technology Research Center of Chinese Academy of Agricultural Sciences, Lanzhou, 730050 China; 3 Frasergen Bioinformatics, Wuhan, 430075 China

**Keywords:** Genome, Genomics

## Abstract

Guide Black-Fur sheep (GD) is a breed of Tibetan sheep (*Ovis aries*) that lives in the Qinghai–Tibetan plateau region at an altitude of over 4,000 m. However, a lack of genomic information has made it difficult to understand the high-altitude adaptation of these sheep. We sequenced and assembled the GD reference genome using PacBio, Hi-C, and Illumina sequencing technologies. The final assembled genome size was 2.73 Gb, with a contig N50 of 20.30 Mb and a scaffold N50 of 107.63 Mb. The genome is predicted to contain 20,759 protein-coding genes, of which 98.42 have functional annotations. Repeat elements account for approximately 52.2% of the genomic landscape. The completeness of the GD genome assembly is highlighted by a BUSCO score of 93.1%. This high-quality genome assembly provides a critical resource for future molecular breeding and genetic improvement of Tibetan sheep.

## Background & Summary

Tibetan sheep play a unique and essential role in China’s rural revitalization strategy. High-quality development of the Tibetan sheep industry is expected to improve social and economic development while maintaining the ecosystem stability and national security of the Qinghai–Tibetan Plateau. Germplasms of these sheep are considered valuable because of their unique biological characteristics, high genetic stability, stress resistance, meat quality, and carpet wool quality^1–3^. Additionally, having undergone long-term natural and artificial selection under specific ecological conditions, they are a model species for studying adaptations to extreme environments. Therefore, assembly of the Tibetan sheep reference genome at the chromosome level will substantially facilitate comparative and functional genomics research. Although draft genome assemblies of certain sheep species have been released, their quality metrics were low^4–8^. Furthermore, no comprehensive genomes of sheep from high-altitude environments (>4,000 m) are available.

Guide Black-Fur sheep (GD) are known for their black skins and spend most of the year grazing in their core habitat of Guide County, Hainan Tibetan Autonomous Prefecture of Qinghai, China (altitude ~4,100 m). GD sheep exhibit a variety of fleece colors, ranging from blackish-red to grey. Black skin contains more melanocytes than other skin colors and thus synthesizes more melanin, absorbing ultraviolet radiation and thereby reducing its damaging effects on organs^9,10^. This adaptation strategy has allowed GD to thrive in a robust ultraviolet environment.

In this study, we aimed to produced high-quality, chromosome-scale, *de novo* genome assemblies of the high-altitude environments GD using Illumina, PacBio, and chromosome conformation capture (Hi-C) sequencing. Comparisons with other mammalian genomes helped to identify rapidly evolving genes and elucidate the evolutionary history of Tibetan sheep.

## Methods

### Sample collection and sequencing

All animal experiments were performed under the guidance of ethical regulations from the Institutional Animal Care and Use Committee of Lanzhou Institute of Husbandry and Pharmaceutical Science of Chinese Academy of Agricultural Sciences (Approval No. NKMYD201805; Approval Date: 18 October 2018). We randomly selected one adult male GD (age: 3 years) from flocks raised under standard feeding regimes and with free access to water in Hainan Tibetan Autonomous Prefecture, Qinghai, China (altitude: ~4,100 m). Blood samples were collected from the jugular vein and then preserved in EDTA anti-freezing tubes at −20 °C. The animals was slaughtered by exsanguination after being deprived of food for 24 h. The brain, heart, liver, kidney, spleen, lung, and muscle tissues were excised within 30 min and stored in liquid nitrogen.

For PacBio long-read and short-read sequencing, genomic DNA was extracted from the blood and liver of the GD. The DNA was sequenced at Frasergen Bioinformatics (Wuhan, China) using the PacBio Sequel platform (Pacific Biosciences), yielding a total of 267.82 Gb of PacBio continuous long reads for corresponding to 98 × genomic coverage (Table 1). The DNA was re-sequenced using HiSeq X-Ten (Illumina, CA, USA) to correct long reads. Paired-end sequencing produced 263.48 Gb of short-read data corresponding to 96 × genomic coverage (Table 1).

**Table 1:** Sequencing data statistics.

Library type	Platform	Application	Tissue	Total bases (bp)	Coverage (X)
Short reads	HiSeqX-Ten	Genome survey and genomic base correction	Blood and liver	263.48	96,499,855.96
Long reads	PacBio Sequel	Genome assembly	Blood and liver	267.82	98,092,157.88
Iso-seq	PacBio Sequel	Annotation	brain, femoral, spleen, back, kidney, heart, lung and liver	36.90	\
Hi-C	HiSeq X-Ten	Chromosome construction	liver	494.40	181,077,741.91

The PacBio Iso-Seq method sequenced full-length transcripts via Single Molecule Real-Time (SMRT) sequencing technology. Total RNA was extracted from all tissues (brain, femur, spleen, back muscle, kidney, heart, lung, and liver) using the RN33 kit (Aidlabs Biotechnologies, Beijing, China). All required profiles were obtained using the same sequencing platform described for PacBio, producing 36.90 Gb of Iso-Seq reads (Table 1).

For Hi-C sequencing, fresh liver tissues were collected for library construction, which was performed as previously described^11^. Paired-end (2 × 150 bp) sequencing was conducted on the Illumina HiSeq X-Ten platform to abtain 494.40 Gb of data corresponding to 181 × genomic coverage (Table 1).

### Genome assembly and polishing

All subreads from SMRT sequencing were used for *de novo* assembly of the GD genome. The draft genome assembly was obtained using MECAT2 with default parameters^12^. Variant calling with gcpp in the SMRT link 4 toolkit was performed to correct errors after the initial genome assembly. Next, initial assembly contigs from the previous step were mapped with corresponding HiSeq reads and polished once using Pilon (v1.22) to correct any remaining errors^13^. This gave a total of 1,972 primary contigs for GD, with an N50 size of 20.30 Mb (Table 2).

**Table 2:** Assembly statistics of Guide black fur sheep.

Assembly	GD
Total length (Mb)	2,730.33
Number of contigs	1,972
Contigs N50 (bp)	20,303,496
Number of scaffolds	726
Scaffold N50 (bp)	107,633,389
Number of gaps	1,246
Total placed scaffolds length (bp)	2,694,746,666 (98.72%)

### Hi-C scaffolding

**Fig. 1:**
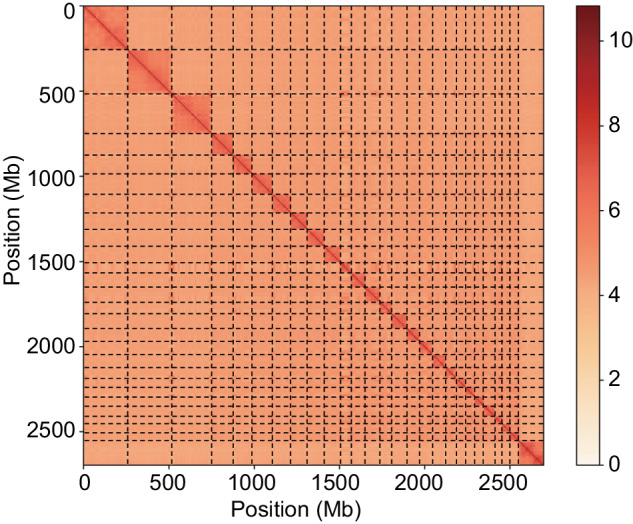
Hi-C interaction heatmap for the GD genome. Color shading from light to dark indicates the increase in interaction intensit.

Next-generation sequencing short reads were aligned to their assembly using the Burrows-Wheeler Aligner (BWA-MEM algorithm, v0.7.17) to further increase single base accuracy^14^. Purge Haplotigs was used to filter redundant sequences resulting from genome heterozygosity^15^. Pseudochromosomes were dcreated during Hi-C analysis, as described previously^16^. Briefly, all clean read pairs produced from the Hi-C library were mapped to the polished sheep contig assembly using the BWA-MEM algorithm with default parameters. LACHESIS was then used to cluster contigs into chromosome-level scaffolds based on the genomic proximity signal of Hi-C data^17^. After scaffolding, we obtained 27 chromosomes containing > 98% of the contigs (Table 3, Fig. 1). The final assembled genome (2.73 Gb) had a scaffold N50 of 107 Mb and 1,246 gaps in total (Table 2).

**Table 3:** Summary of the Hi-C grouping results in each pseudo chromosome.

Superscaffold	Number of contigs	Total length
Superscaffold1	66	258,182,173
Superscaffold2	85	257,143,650
Superscaffold3	100	234,249,426
Superscaffold4	55	125,419,520
Superscaffold5	32	110,522,948
Superscaffold6	55	122,463,329
Superscaffold7	86	107,590,889
Superscaffold8	37	93,936,628
Superscaffold9	58	100,738,854
Superscaffold10	43	92,692,752
Superscaffold11	18	63,722,430
Superscaffold12	78	84,668,624
Superscaffold13	36	85,589,744
Superscaffold14	31	69,024,306
Superscaffold15	48	86,505,251
Superscaffold16	81	78,983,106
Superscaffold17	7	73,557,225
Superscaffold18	97	77,856,120
Superscaffold19	36	63,428,258
Superscaffold20	28	53,156,399
Superscaffold21	18	52,734,379
Superscaffold22	30	53,558,222
Superscaffold23	20	63,901,777
Superscaffold24	32	44,738,995
Superscaffold25	33	47,433,049
Superscaffold26	18	45,735,537
Superscaffold27	45	147,213,075
Total placed	1,273	2,694,746,666

### Annotation of repetitive sequences and genes

*De novo* and homology-based prediction methods annotated repeat sequences in the GD genome. Known transposable elements were identified by combining RepeatMasker, RepeatProteinMask, and RepeatModeler^18^. Repetitive transposable element (TE) sequences comprised ~52% of the total assembly of the genome, with long terminal repeat retrotransposons being the most abundant (~20.99%) (Table 4).

**Table 4:** Summary of genome repeats in the GD genome.

Type	Repeat size (bp)	% of Genome
DNA	105,509,297	3.86
LINE	1,199,308,906	43.93
SINE	47,731,898	1.75
LTR	573,144,758	20.99
Other	0	0
Unknown	35,477,461	1.3
Total TE	1,425,306,254	52.2

Note: DNA, DNA transposon; LINE, long interspersed nuclear elements (>1,000 bp); SINE, short interspersed nuclear elements (~300 bp); LTR, long terminal repeats (1.5–10 kbp); TE, repetitive transposable element.

**Fig. 2:**
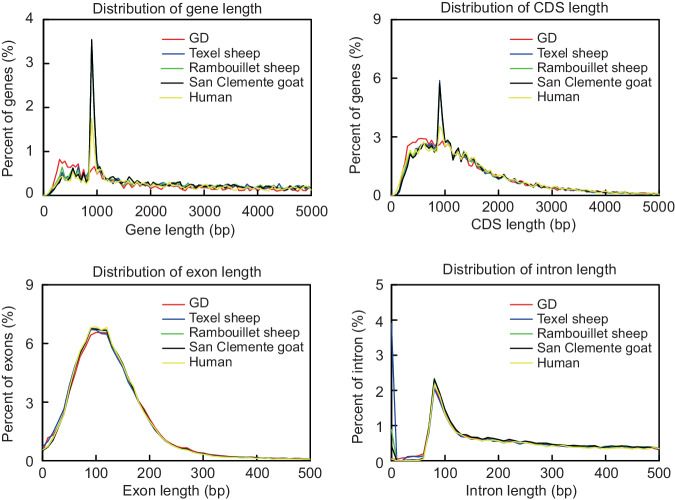
Comparison of gene features among the genomes of GD and four other animal species. Gene features include the lengths of genes, CDS, exons, and introns.

For gene annotation, we adopted a strategy combining *ab initio* gene finding, homology-based gene prediction, and Iso-Seq reads. First, the assembled GD genome was hard- and soft- masked by RepeatMasker. Second, *ab initio* gene prediction was performed using the Augustus (v3.3.1) and SNAP gene models, which were pre-trained using homologous proteins^19^. Third, Exonerate (v2.2.0) set to default parameters was used to predict genes from protein sequences^20^. Fourth, clean RNA-sequencing reads were assembled into transcripts via Trinity for RNA-based gene prediction, followed by further prediction of gene structure using PASA^21^. Finally, Maker (v3.00) was employed to integrate the three sets of prediction results^22^. In total, we identified 20,759 predicted protein-coding genes within the genome, representing 98.42% of all genes (Table 5). A comparison of gene features among GD, Texel sheep (*Ovis aries*)^23^, Rambouillet sheep (*Ovis aries*)^24^, San Clemente goats (*Capra hircus*)^25^, and humans (*Homo sapiens*)^26^ revealed similar length distributions for coding sequences, genes, exons, and introns (Fig. 2).

**Table 5:** Functional annotation statistics of the GD genome.

Type	Number	Percentages(%)
Total	20759	\
InterPro	18419	88.73
GO	13972	67.31
KEGG_ALL	20289	97.74
KEGG_KO	13611	65.57
Swissprot	19862	95.68
TrEMBL	20166	97.14
NR	20393	98.24
Annotated	20432	98.42
Unannotated	327	1.58

Note: GO, Gene Ontology; KEGG, Kyoto Encyclopedia of Genes and Genomes; NR, non-redundant.

The tRNA-related genes were mainly identified by tRNAscan-SE (v1.3.1) and Infernal (v1.1.2) using default parameters^18,27^. A total of 256,815 non-coding RNA genes were predicted, comprising 528 miRNA, 231 rRNA, 264,021 tRNA, and 2,017 snRNA genes (Table 6).

**Table 6:** Information on non-protein-coding genes identified in the GD genome.

Type		Copy	Average length (bp)	Total length (bp)	% of genome
miRNA		528	85.24	45,005	0.0016
tRNA		264,021	73.26	19,341,134	0.7025
rRNA	18s	34	1546.76	52,590	0.0019
	28s	26	3206.27	83,363	0.0030
	5.8s	30	154.23	4,636	0.0002
	5s	95	101.35	9,628	0.0003
	8s	0	0.00	0	0.0000
snRNA	CD-box	274	90.48	24,791	0.0009
	HACA-box	400	131.67	52,667	0.0019
	splicing	1,339	110.89	148,478	0.0054
	scaRNA	28	158.25	4,431	0.0002

## Data Records

Raw data from the long-read and short-read sequencing have been deposited in the NCBI Sequence Read Archive database with accession numbers SRR22290763 (Iso-seq) and SRR22585187 (whole-genome sequencing) under BioProject PRJNA898852^28,29^. The final chromosome assembly was deposited in the GenBank at NCBI with accession number: JBEJUG010000000^30^. The draft genome assembly and genome annotation were deposited in the *Figshare* database (10.6084/m9.figshare.26013145)^31^.

## Technical Validation

### Evaluation of the genome assembly

Quality metrics included assessment of the completeness of each genome based on the proportion of single-copy conserved orthologs obtained and specific signs of mis-assembly. Benchmarking Universal Single-Copy Orthologs (BUSCO) software^32^ revealed that ~93% of the conserved genes were identified in the GD genome, confirming the completeness of the obtained assemblies. The 93.1% of single-copy conserved orthologs in this assembly was higher than those found in Hu sheep and Texel sheep^23,33^. The percentage of duplicated complete BUSCOs detected in the GD genome (1.9%) was comparable to the ranges observed across the genomes of Texel sheep and Rambouillet sheep (0.9%–1.6%)^23,24^. The GD genome also exhibited a lower percentage of missing BUSCOs (4.2%) compared with the Texel sheep genome (11.1%)^23^, suggestive of genomic integrity and the precise assembly of highly complex regions (Table 7).

**Table 7:** BUSCO completeness analysis of genome assembly.

Type	Proteins	Percentages(%)
Complete BUSCOs	3,823	93.1
Complete and single-copy BUSCOs	3,743	91.2
Complete and duplicated BUSCOs	80	1.9
Fragmented BUSCOs	109	2.7
Missing BUSCOs	172	4.2
Total BUSCO groups searched	4,104	100

### Genome collinearity analysis

**Fig. 3:**
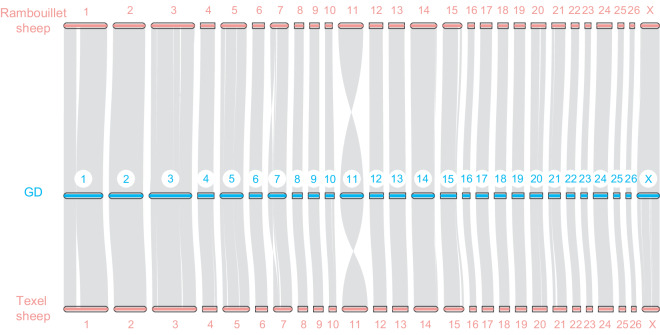
Genome collinearity among the GD, Texel, and Rambouillet sheep breeds.

Genome synteny analysis of the GD, Texel, and Rambouillet sheep breeds was performed using the MUMmer tool with default parameters (filtering of delta sequences with the -1 parameter and removal of collinear fragments <10 kb). Single-nucleotide polymorphisms and variations between the three genomes were identified using the show-snps (-rT parameter) and show-diff (-rH parameter) utilities, respectively. Overall, the GD genome demonstrates strong collinearity with Texel and Rambouillet sheep. Some obvious insertions and inversions on chromosomes 1, 3, and 7 may be species-specific (Fig. 3).

## Data Availability

No specific code was developed in this work. The parameters of all commands and pipelines used for data processing are described in the Methods section. If no detailed parameters are mentioned for a software, the default parameters were used, as suggested by the developer.

## References

[1] Liu JB (2020). Genetic signatures of high-altitude adaptation and geographic distribution in Tibetan sheep. Sci Rep..

[2] Zhang QY (2021). Gangba sheep in the Tibetan plateau: validating their unique meat quality and grazing factor analysis. J Environ Sci..

[3] Liu GB (2013). Identification of microRNAs in wool follicles during anagen, catagen, and telogen phases in Tibetan sheep. PloS One.

[4] Davenport KM (2022). An improved ovine reference genome assembly to facilitate in-depth functional annotation of the sheep genome. GigaScience.

[5] Jiang Y (2014). The sheep genome illuminates biology of the rumen and lipid metabolism. Science.

[6] Li R (2021). A Hu sheep genome with the first ovine Y chromosome reveal introgression history after sheep domestication. Sci China Life Sci.

[7] Upadhyay M (2020). The first draft genome assembly of snow sheep (*Ovis nivicola*). Genome Biol. Evol..

[8] Yang YZ (2017). Draft genome of the Marco Polo Sheep (*Ovis ammon polii*). GigaScience.

[9] Li MZ (2013). Genomic analyses identify distinct patterns of selection in domesticated pigs and Tibetan wild boars. Nat Genet.

[10] Visscher MO (2017). Skin color and pigmentation in ethnic skin. Facial Plast Surg Clin North Am..

[11] Crémazy FG (2018). Determination of the 3D genome organization of bacteria using Hi-C. Methods Mol Biol..

[12] Xiao CL (2017). MECAT: fast mapping, error correction, and de novo assembly for single-molecule sequencing reads. Nat Methods.

[13] Walker BJ (2014). Pilon: an integrated tool for comprehensive microbial variant detection and genome assembly improvement. PloS One.

[14] Li, H. Aligning sequence reads, clone sequences and assembly contigs with BWA-MEM. *arXiv preprint* **1303**, 3997 (2013).

[15] Roach MJ, Schmidt SA, Borneman AR (2018). Purge haplotigs: allelic contig reassignment for third-gen diploid genome assemblies. BMC Bioinf..

[16] Yin DM (2018). Genome of an allotetraploid wild peanut Arachis monticola: a de novo assembly. GigaScience.

[17] Burton JN (2013). Chromosome-scale scaffolding of de novo genome assemblies based on chromatin interactions. Nat Biotechnol.

[18] Tarailo-Graovac M, Chen N (2009). Using RepeatMasker to identify repetitive elements in genomic sequences. Curr. Protoc. Bioinf..

[19] Stanke M (2006). AUGUSTUS: ab initio prediction of alternative transcripts. Nucleic Acids Res..

[20] Slater GS, Birney E (2005). Automated generation of heuristics for biological sequence comparison. BMC Bioinf..

[21] Grabherr MG (2011). Full-length transcriptome assembly from RNA-Seq data without a reference genome. Nat Biotechnol.

[22] Cantarel BL (2008). MAKER: an easy-to-use annotation pipeline designed for emerging model organism genomes. Genome Res..

[23] (2015). NCBI GenBank.

[24] (2017). NCBI GenBank.

[25] (2016). NCBI GenBank.

[26] (2022). NCBI GenBank.

[27] Nawrocki EP, Kolbe DL, Eddy SR (2009). Infernal 1.0: inference of RNA alignments. Bioinformatics.

[28] (2023). NCBI Sequence Read Archive.

[29] (2023). NCBI Sequence Read Archive.

[30] (2024). NCBI GenBank.

[31] Lu ZK (2024). figshare..

[32] Simão FA, Waterhouse RM, Ioannidis P, Kriventseva EV, Zdobnov EM (2015). BUSCO: assessing genome assembly and annotation completeness with single-copy orthologs. Bioinformatics.

[33] (2020). NCBI GenBank.

